# Active Constituents and Mechanisms of Xinshubao Tablets in Coronary Vasorelaxation

**DOI:** 10.3390/ph19050704

**Published:** 2026-04-29

**Authors:** Zhenkun Li, Hongwei Wu, Wenjie Li, Bo Zhang, Shengxuan Cao, Qingqing Cai, Hongjun Yang

**Affiliations:** 1 Institute of College of Pharmacy, Changchun University of Chinese Medicine, Changchun 130117, China; 2 Institute of Chinese Materia Medica, China Academy of Chinese Medical Sciences, Beijing 100700, China; 3 Institute of Basic Theory for Chinese Medicine, China Academy of Chinese Medical Sciences, Beijing 100700, China; 4 Institute of Traditional Chinese Medicine Health Industry, China Academy of Chinese Medical Sciences, Nanchang 330115, China; 5 Experimental Research Center, China Academy of Chinese Medical Sciences, Beijing 100700, China

**Keywords:** Xinshubao tablet, *Crataegus pinnatifida* Bge, isochlorogenic acid B, vasodilation, intestinal absorption solutions

## Abstract

**Background:** Xinshubao tablet (XSB), a traditional Chinese medicine (TCM) formula composed of five medicinal herbs, is used clinically to alleviate cardiovascular diseases. This study aimed to investigate the coronary vasodilatory effects of XSB and its individual herbs, exploring its active constituents and the underlying mechanisms. **Methods:** The vasorelaxant effects of XSB and its individual herbal intestinal absorption solutions (IASs) were evaluated by ex vivo coronary artery ring assays. The chemical constituents of the best active herbal IAS were qualitatively identified using ultra-performance liquid chromatography–quadrupole time-of-flight mass spectrometry (UPLC–Q-TOF-MS). Molecular docking and ex vivo assays were used to predict and validate the bioactive constituents and mechanisms responsible for coronary vasorelaxation. **Results:** Vasodilation experiments revealed that XSB-IAS and its individual herb IAS exhibited varying degrees of vasodilatory effects, in the range of 0.8–18 g raw materials/mL. At 6, 12, and 18 mg of raw materials/mL, *Crataegus pinnatifida* Bge (Shanzha) exhibited vasodilation rates of 26.45% ± 1.8%, 36.57% ± 3.5%, and 45.16% ± 6.3%, which were obviously higher than those of the other individual herbs. Fifty constituents in Shanzha IAS were identified by UPLC-Q-TOF-MS. Vasodilation-related protein–protein interaction (PPI) network revealed NOS3 as a core regulatory target. Molecular docking demonstrated that among the identified constituents, isochlorogenic acid B, betulin, etc., displayed binding affinity to NOS3. Isochlorogenic acid B was further validated to exhibit vasodilatory effects in the ranges of 0.05–2.5 mM. Mechanistic results showed that isochlorogenic acid B improved vasodilation by inhibiting Ca^2+^ influx through both voltage-dependent and receptor-operated Ca^2+^ channels, activating K^+^ channels, and exhibiting endothelium-dependent vasorelaxation. **Conclusions:** This study provides insights into the material basis and mechanisms underlying the vasorelaxant effects of XSB. Isochlorogenic acid B was firstly found to exert the coronary vasodilatory effect. This study can also contribute to the identification of efficacy-related quality markers in TCM.

## 1. Introduction

Traditional Chinese medicine (TCM) formulae are characterized by multi-component, multitarget, and multi-pathway effects. Unlike single compound chemical drugs, TCM formulae exhibit synergistic therapeutic effects and reflect the holistic perspective and the principle of syndrome differentiation and treatment [1]. Nevertheless, these advantages are accompanied by considerable complexity. The chemical diversity of TCM formulae poses significant challenges for elucidating their pharmacologically active material basis. Furthermore, the molecular targets and signaling pathways through which the bioactive constituents exert their effects remain major bottlenecks in current research [2].

Myocardial infarction (MI) is a major cause of mortality worldwide [3]. Its incidence continues to increase in line with population aging and the increasing prevalence of risk factors such as smoking, hypertension, diabetes, and dyslipidemia [4]. Current pharmacological interventions mainly include antiplatelet agents (e.g., aspirin), anticoagulants (e.g., heparin), β-receptor blockers, statins, and vasodilators [5]. Among these, vasodilators play a pivotal role in enhancing coronary blood flow, reducing myocardial oxygen consumption, and alleviating ischemic injury [6]. Vasodilators exert their effects via multiple mechanisms. For example, some calcium channel blockers inhibit Ca^2+^ influx and decrease vascular smooth muscle contraction [7]. Potassium (K^+^) channel openers induce cellular hyperpolarization, indirectly suppressing Ca^2+^ channel activity and promoting vasorelaxation [8]. Endothelium-dependent vasodilators act by stimulating the release of nitric oxide (NO), carbon monoxide (CO), and prostacyclin, enhancing relaxation responses [9]. These mechanisms synergistically reduce the cardiac afterload and enhance coronary perfusion, constituting a central strategy for treating MI.

XSB, composed of *Salvia miltiorrhiza* Bunge (Danshen), *Paeonia lactiflora* Pall. (Baishao), *Curcuma kwangsiensis* S. G. Lee et C. F. Liang (Yujin), *Acanthopanax senticosus* (Rupr. et Maxim.) Harms (Ciwujia), and *Crataegus pinnatifida* Bunge (Shanzha), promotes blood circulation, removes blood stasis, tonifies qi, and relieves pain. Clinically, it is mainly prescribed to treat coronary heart disease and alleviate symptoms such as chest tightness and angina associated with qi deficiency and blood stasis syndromes [10,11,12]. Previous studies have reported the vasorelaxant effects of XSB on mesenteric vessels; however, mesenteric vessels primarily participate in regulating peripheral resistance and blood flow distribution, whereas coronary arteries are responsible for cardiac perfusion and balancing the oxygen supply. Given the fundamental differences in their physiological roles, it is therefore necessary to further investigate the effects of XSB on the coronary vascular system [13]. Therefore, this study aimed to investigate the coronary vasodilatory effects of XSB and identify its active constituents and underlying mechanisms.

Intestinal absorption solutions (IASs), prepared using the everted gut sac method serve as a suitable ex vivo pharmacological approach for the evaluation of traditional Chinese medicine (TCM). This method not only eliminates the influence of nonabsorbed constituents on pharmacological effects but also avoids interference from endogenous substances present in drug-containing serum. Moreover, compared with the original herbal materials, IASs contain fewer components and are present at higher concentrations than those detected in systemic circulation, thereby reducing analytical complexity and facilitating the identification of bioactive constituents [14]. But IAS also has certain limitations. For instance, it cannot adequately reflect the influence of intestinal, bile, and other luminal factors on drug absorption. In addition, the viability of the intestinal segments gradually declines over time which may affect the reliability of the experimental results. Therefore, experiments using this model are generally conducted within 3 h at 37 °C under continuous aeration (95% O_2_ and 5% CO_2_) to maintain tissue viability [15]. Despite these inherent limitations associated with the model itself, the IAS remains a rapid and suitable approach for ex vivo pharmacodynamic evaluation and active component screening of traditional Chinese medicine (TCM) formulas.

**Figure 1:**
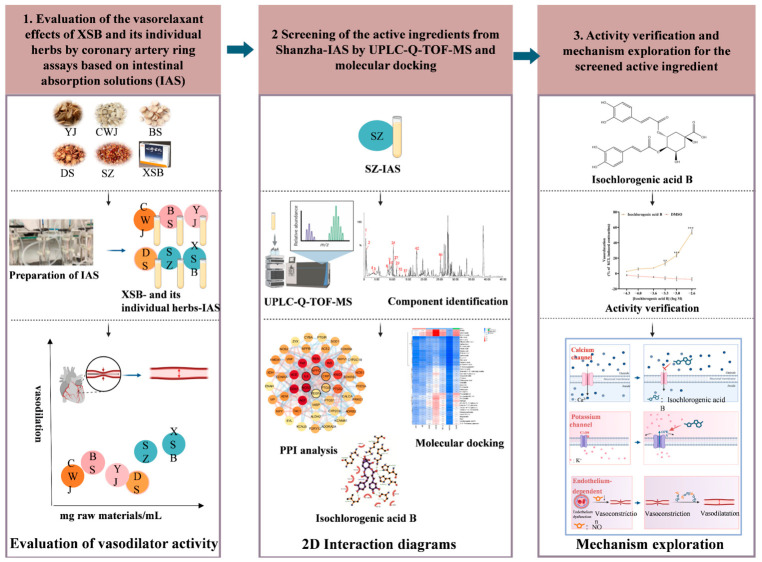
Schematic workflow for identifying effective components and underlying mechanisms of XSB in vasodilation.

Ex vivo coronary artery ring assays showed that XSB and single-herb IAS demonstrated vasorelaxant activity, with Shanzha IAS showing the most pronounced effect. Subsequently, Shanzha IAS was profiled using ultra-performance liquid chromatography–quadrupole time-of-flight mass spectrometry (UPLC-Q-TOF-MS), and a comprehensive chemical fingerprint was established. Molecular docking analysis [16], based on the identified compounds, was used to discover the active components. Findings from ex vivo experiments revealed the coronary vasodilatory effect of isochlorogenic acid B, which is a novel finding reported in this study. This vasorelaxant activity was mediated through the modulation of Ca^2+^ channels, K^+^ channels, and endothelium-dependent pathways (Figure 1).

In summary, by using a progressive strategy of integrating pharmacological screening, component identification, molecular docking, and mechanistic validation, this study systematically elucidates the material basis of XSB at the formula, single-herb, and compound levels. This research approach offers a novel and feasible method to analyze the complex active constituents and mechanisms of action in TCM formulas. Crucially, guided by this research approach, isochlorogenic acid B was identified for the first time as possessing potential cardiovascular vasodilatory activity. This study not only demonstrates the viability of the aforementioned research approach but also provides a scientific basis for the development of novel vasodilators.

## 2. Results and Discussion

### 2.1. XSB and Its Single-Herb IAS Can Dilate Coronary Blood Vessels

**Figure 2:**
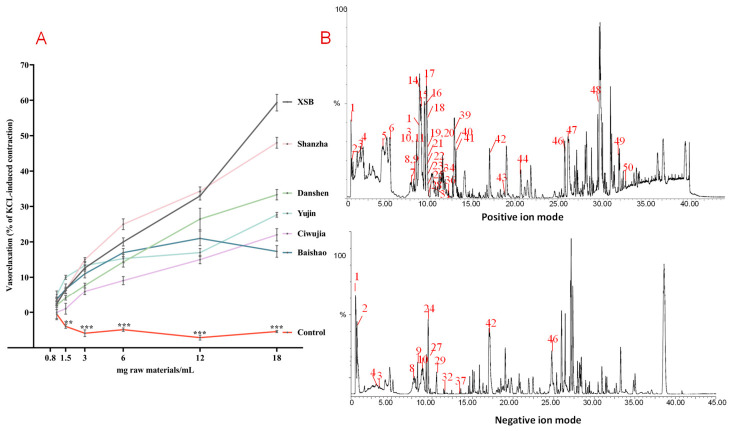
Vasorelaxant activity evaluation of XSB and its single-herb IAS, and qualitative analysis of Shan zha IAS. The results are presented as means ± SD (*n* = 6 coronary artery rings). (**A**) Vasodilatory effects of XSB and its single-herb IAS on the coronary artery. The *X*-axis represents concentration of intestinal absorption fluid: 0.8–18 mg of raw materials/mL. The *Y*-axis represents vasorelaxation. ** p < 0.01, and *** p < 0.001. The control group vs. all the drug groups. (**B**) Representative base peak chromatograms of Shanzha IAS. Positive ion mode and negative ion mode. The ingredients corresponding to the numbers in the figure are listed in Table S1.

XSB, as a TCM compound preparation, exhibited vasodilatory effects in isolated rat coronary arteries, showing a concentration-dependent vasorelaxation with significantly higher rates (63.7% ± 4.03%, *p* < 0.001) compared with the control group. Further comparisons of single herbal solution IAS (Danshen: 36.04% ± 5.74%, *p* < 0.001; Baishao: 19.75% ± 2.24%, *p* < 0.001; Ciwujia: 22.81% ± 5.61%, *p* < 0.001; Yujin: 27.33% ± 6.32%, *p* < 0.001) revealed that all single herbs exerted vasodilation effects, with Shanzha showing the most potent activity. A vasorelaxation rate of 40.85% ± 2.92%, *p* < 0.001, was achieved at a concentration of 18 mg of raw materials/mL (Figure 2A).

Shanzha is the mature fruit of C pinnatifida Bge. var. major N.E.Br. or C. Pinnatifida Bge. [17]. Shanzha is rich in flavonoids, organic acids, and triterpenoids. Previous studies have reported that Shan zha possesses multiple cardiovascular protective effects, including antihypertensive, lipid-lowering, anti-atherosclerotic, and anti-arrhythmic activities [18]. In this part, we have confirmed that XSB also has a vasodilatory effect on coronary arteries, with Shanzha likely serving as a major active contributor. 

### 2.2. Chemical Profiling of ShanZha IAS Using UPLC-Q-TOF-MS

UPLC-Q-TOF-MS was performed in both positive and negative ion full-scan modes to comprehensively characterize the chemical profile of Shanzha IAS, and the corresponding base peak chromatograms were obtained (Figure 2B). By referencing databases and combining accurate molecular masses with characteristic fragment ions, a total of 50 compounds were successfully identified in the Shanzha IAS (Supplementary Materials). In the strategy employed in this study, to identify as many chemical constituents as possible, we first used Q-TOF-MS for the comprehensive analysis and identification of components in the IAS. Active components were then predicted from the identified compounds using molecular docking, and their activities were ultimately verified with selected reference standards. This approach avoids the need to obtain a large number of reference substances and conduct methodological evaluations for quantitative analysis at the initial stage of the experiment. Meanwhile, it should be noted that concentration serves as an important reference for screening active ingredients and holds significance for subsequent in vivo experiments.

### 2.3. Screening of Bioactive Compounds Based on Molecular Docking

**Figure 3:**
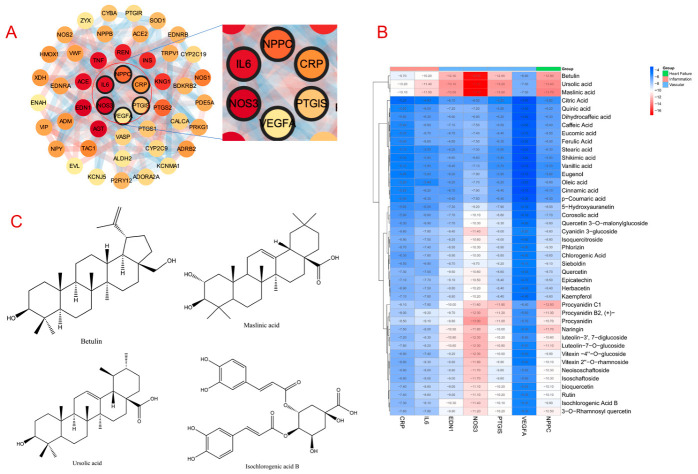
Screening key vasodilator targets and molecular docking heatmaps of small-molecule compounds with target proteins (CRP, IL-6, EDN1, NOS3, PTG1, VEGFA, NPPC). (**A**) Identification of the core proteins associated with vascular relaxation using PPI analysis. The redder the color of the circle, the stronger the interaction with other targets. (**B**) Heatmap of molecular docking scores between Shanzha IAS compounds and vasodilation targets. The “Group” annotation is used to categorize the vasodilation-related target genes (columns) into functional modules for visualization and biological interpretation. Docking results of 50 compounds with seven protein targets (NOS3, EDN1, PTGIS, NPPC, VEGFA, CRP, and IL6) are presented. Color gradients range from red (high affinity, binding energy < –12 kcal/mol) to blue (low affinity, binding energy > –6 kcal/mol). Bidirectional hierarchical clustering (Euclidean distance) of compounds and targets is shown in the left and top dendrograms. (**C**) Chemical structures of betulin, maslinic acid, ursolic acid, and isochlorogenic acid B.

The core targets related to vasodilation were retrieved from the Gene cards database using “vasodilation” as a keyword. The top 50 targets ranked by relevance score were imported into the STRING database. PPI analysis revealed natriuretic peptide C (NPPC), C-reactive protein (CRP), Prostaglandin I2 synthase (PTGIS), vascular endothelial growth factor A (VEGFA), NOS3, and interleukin (IL-6) to be highly connected to other proteins. These proteins may represent key regulators involved in modulating vascular relaxation (Figure 3A).

Based on high throughput virtual screening, each constituent of Shanzha IAS was individually docked with the target proteins (NPPC, CRP, PTGIS, VEGFA, NOS3, and IL-6), yielding a total of 300 binding energy data points (50 × 6). The docking scores were normalized, and bidirectional hierarchical clustering together with heatmap visualization was performed using the heatmap package in R. Clustering analysis revealed that several compounds exhibited strong binding affinities with the five target proteins, with particularly pronounced interactions noted for NOS3. Specifically, betulin (−16.80 kcal/mol), ursolic acid (−17.30 kcal/mol), maslinic acid (−17.40 kcal/mol), and isochlorogenic acid B (−11.40 kcal/mol) showed favorable binding to NOS3 (Figure 3B).

NOS3 is predominantly expressed in vascular endothelial cells and catalyzes the coversion of L-arginine to NO. As a key endothelium-dependent vasodilator, NO activates soluble guanylate cyclase, leading to elevated cyclic guanosine monophosphate (cGMP) levels and ultimately leading to vascular smooth muscle relaxations. Therefore, NOS3 is crucial in maintaining vasodilation, blood perfusion, and blood pressure homeostasis. Moreover, NOS3 is not only a central hub for the synthesis of vasodilatory factors but is also an important link between inflammatory injury and vascular dysfunction. The upregulation of NOS3 expression in cardiomyocytes by XSB was also demonstrated in our previous study [13].

**Figure 4:**
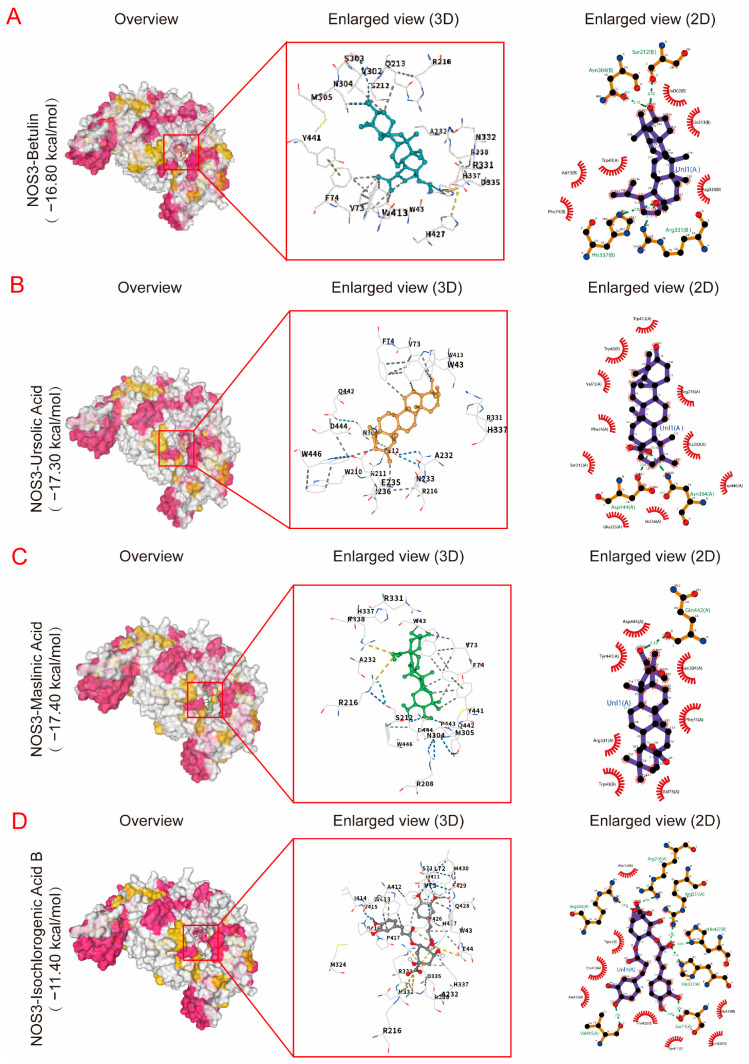
Representative molecular docking conformations and interaction modes of small molecules with NOS3. Docking structures of botulin (**A**), ursolic acid (**B**), maslinic acid (**C**), and isochlorogenic acid B (**D**) with NOS3 are shown. (**Left**) Whole protein with ligand localization (overview); (**Middle**) magnified 3D active pocket highlighting hydrogen bonds, hydrophobic interactions, and π–π stacking; (**Right**) 2D interaction diagrams illustrating the noncovalent interaction network. Isochlorogenic acid B forms multiple hydrogen bonds and hydrophobic contacts, suggesting strong binding stability and potential modulation of NOS3 activity.

To further clarify the mode of interaction between small molecules and the vasodilator targets, betulin (Figure 4A), ursolic acid (Figure 4B), maslinic acid (Figure 4C), and isochlorogenic Acid B (Figure 4D) having strong docking affinity, were selected for the construction of ligand–receptor complex models with the NOS3 protein. Each component exhibited a certain docking ability with NOS3. Currently, there are no reports demonstrating the vasorelaxant effect of betulin. However, betulin and betulinic acid are related as a parent compound and its oxidized derivative. Previous studies have shown that betulinic acid can increase NOS3 phosphorylation and NO synthesis via the calcium-signaling pathway [19]. Ursolic acid is a pentacyclic triterpenoid compound. Ex vivo studies have shown that ursolic acid can dilate blood vessels via both endothelium-dependent pathways, including enhanced endothelial NO production and inhibition of voltage-dependent Ca^2+^ channels [20]. Studies also have reported that maslinic acid, a pentacyclic triterpenoid, induces concentration-dependent vasorelaxation in aortic rings isolated from spontaneously hypertensive rats, suggesting a direct role in the regulation of vascular tone [21].

Isochlorogenic acid B displays the strongest docking affinity. Isochlorogenic acid B is one of the flavonoids present in Shanzha. Modern pharmacological studies have demonstrated its diverse biological activities, including its anti-inflammatory [22], antioxidant [23], anxiolytic, and antidepressant effects [24]. However, the vasorelaxant effects of isochlorogenic acid B have not been reported to date. In our study, it formed up to seven hydrogen bonds with residues including Arg, Glu, and Thr in the active pocket of NOS3. Furthermore, noncovalent interactions with surrounding aromatic hydrophobic residues (e.g., Phe, Tyr) were also observed, indicating a high degree of spatial complementarity with the active site (Figure 4D). These results suggest the stronger binding stability and multipoint interaction profile of isochlorogenic acid B over other compounds for NOS3.

**Figure 5:**
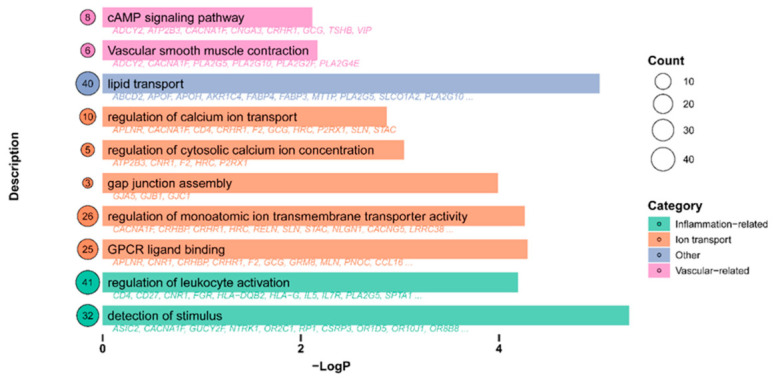
Functional enrichment analysis of the top 1% differentially expressed genes of isochlorogenic acid B from ITCM. (**Left**) The numbers inside the circles represent the number of enriched targets. (**Right**) Classified according to different pathways (inflammation-related, ion transport, vascular-related): the longer the line, the greater the number of enriched targets. Representative targets under each pathway are also indicated.

For elucidation of the potential mechanisms, the top 1% of differentially expressed genes of isochlorogenic acid B from the ITCM database were subjected to enrichment analysis (Figure 5). Significant enrichment of pathways related to ion transport and inflammation was noted, suggesting that isochlorogenic acid B may exert its effects by regulating multiple pathways.

### 2.4. Isochlorogenic Acid B Can Dilate Coronary Vessels

#### 2.4.1. Isochlorogenic Acid B Can Dilate Coronary Artery Rings Pre-Contracted by KCl and U46619

Vasodilation plays an important role in the treatment of MI. It improves coronary blood flow, alleviates myocardial ischemia, and inhibits ventricular remodeling, ultimate-ly providing cardioprotection. Vasodilation is a complex physiological process mediated by multiple pharmacological mechanisms.

**Figure 6:**
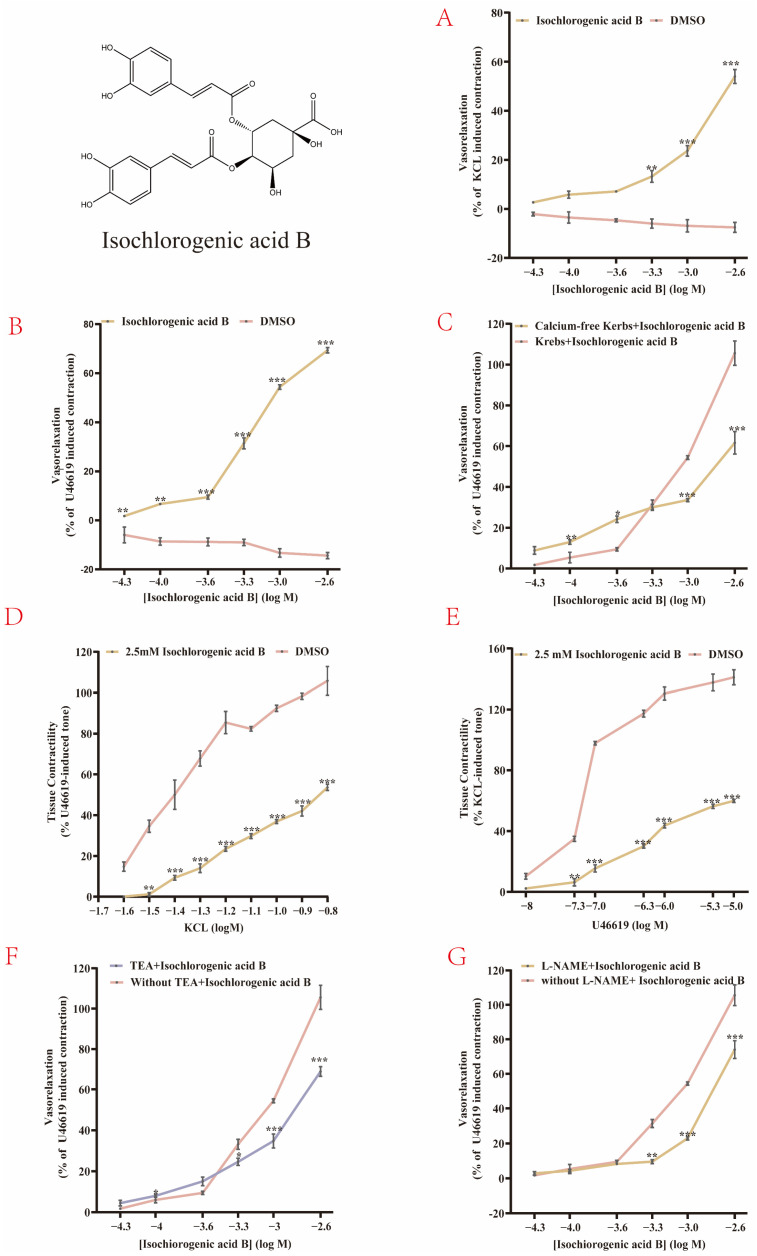
Vasorelaxant effects and underlying mechanisms of isochlorogenic acid B on coronary arterial rings. The results are presented as means ± SD (*n* = 6 coronary artery rings). (**A**) Isochlorogenic acid B induces dose-dependent vasorelaxation in KCl-precontracted coronary artery rings. (**B**) Isochlorogenic acid B induces dose-dependent vasorelaxation in U46619-precontracted coronary artery rings. (**C**) The vasorelaxant effect of isochlorogenic acid B is associated with Ca^2+^ channels. (**D**,**E**) Isochlorogenic acid B may exert its vasorelaxant effect by inhibiting VGCCs and ROCCs. (**F**) The vasorelaxant effect involves activation of K^+^ channels. (**G**) The vasorelaxant effect of isochlorogenic acid B is endothelium-dependent. * *p* < 0.05, ** *p* < 0.01, *** p < 0.001 vs. the control group.

Although molecular docking suggested the potential of isochlorogenic acid B as a vasodilator, no experimental evidence has been reported to date. Therefore, ex vivo experiments were conducted to preliminarily verify its vasodilatory effect and investigate its underlying mechanisms. In the control group, only minimal vasorelaxation was observed in the coronary artery rings that were precontracted with KCl or U46619. The vasorelaxant effect gradually increased with increasing concentrations of isochlorogenic acid B (5 × 10^−5^, 1 × 10^−4^, 2.5 × 10^−4^, 5 × 10^−4^, 1 × 10^−3^, and 2.5 × 10^−3^ mol/L). At 2.5 × 10^−3^ mol/L, the relaxation rate reached (49.29% ± 7.66%, *p* < 0.001) in KCl-precontracted rings (Figure 6A) and (70.53% ± 6.15%, *p* < 0.001) in U46619-precontracted rings (Figure 6B).

On the basis of dose–response curves, the ex vivo EC_50_ values of 0.9 mM (precontracted with KCl) and 2.8 mM (precontracted with U46619) for vasorelaxation are comparable to the concentrations commonly used in isolated vessel assays for flavonoids, polyphenols, and botanical extracts [25]. Based on the EC_50_ values, the corresponding in vivo effective concentration was estimated to be 0.9–28 μM by IVIVE principles. This concentration range is achievable with therapeutic dosing, as reported in in vivo studies of similar vasoactive natural products [26]. Thus, these results not only confirm the vasorelaxant activity of isochlorogenic acid B, but also establish a concentration reference for further in vivo investigations.

#### 2.4.2. Isochlorogenic Acid B Exerts Vasodilatory Effects by Inhibiting Ca^2+^ Influx

Calcium channels occupy a central position in regulating vasodilation, as their functional state directly determines the tension of vascular smooth muscle cells [27]. We examined whether the vasorelaxant effect of isochlorogenic acid B was calcium-dependent and found that the relaxation of U46619-precontracted rings was markedly reduced under calcium-free conditions (58.50% ± 6.76%, *p* < 0.001) (Figure 6C), indicating a calcium-dependent mechanism.

Vasodilation is primarily achieved by reducing the intracellular Ca^2+^ concentration in vascular smooth muscle cells. In particular, inhibition of VGCC- and ROCC-dependent Ca^2+^ channels reduces Ca^2+^ influx, thereby inhibiting vascular smooth muscle contraction and ultimately inducing vasodilation; this is one of the key mechanisms by which vasoactive substances exert effects.

We conducted experiments to verify whether the effects of isochlorogenic acid B are mediated by the aforementioned Ca^2+^ channels. Coronary artery rings pretreated with 2.5 × 10^−3^ mol/L of isochlorogenic acid B for 10 min exhibited significantly reduced contraction in response to cumulative KCl administration compared with the control group. KCl induces vasoconstriction by depolarizing the vascular smooth muscle cell membrane, thereby activating VGCCs and the subsequent reduction in Ca^2+^ influx. The maximal contraction decreased from 106.42% ± 5.86% to 52.53% ± 2.5%, indicating that isochlorogenic acid B may inhibit vasoconstriction by suppressing VGCC-mediated Ca^2+^ influx. (Figure 6D). Similarly, pretreatment with isochlorogenic acid B attenuated U46619-induced contraction, suggesting that it could also suppresses receptor-operated Ca^2+^ entry (Figure 6E).

These findings collectively indicate that its vasodilatory effects may involve inhibition of both VGCC- and ROCC-mediated Ca^2+^ influx, thereby reducing intracellular Ca^2+^ levels and attenuating vascular contraction.

#### 2.4.3. Isochlorogenic Acid B Exerts Vasodilatory Effects by Activation K^+^ Channels

K^+^ channels also play an important role in regulating vascular relaxation. The activation of K^+^ channels induces K^+^ efflux, leading to membrane hyperpolarization, which effectively suppresses the activation of VGCCs, reduces Ca^2+^ influx, and ultimately promotes vascular smooth muscle relaxation [28]. Pretreatment with the nonselective K^+^ channel blocker TEA (1 mM, 10 min) significantly attenuated (67.46% ± 2.96%, *p* < 0.001) the vasorelaxant effect of isochlorogenic acid B compared with the control group (without TEA) (Figure 6F). This finding suggested that isochlorogenic acid B-induced relaxation is partly mediated through K^+^ channel activation, which alters membrane potential and Ca^2+^ entry.

#### 2.4.4. Isochlorogenic Acid B May Exert Endothelium-Dependent Vasodilatory Effects

Moreover, endothelium-dependent relaxation is another critical regulatory mechanism. When endothelial dysfunction occurs (such as atherosclerosis occur), NOS3 uncoupling leads to increased ROS generation, thereby reducing vasorelaxant capacity.

**Figure 7:**
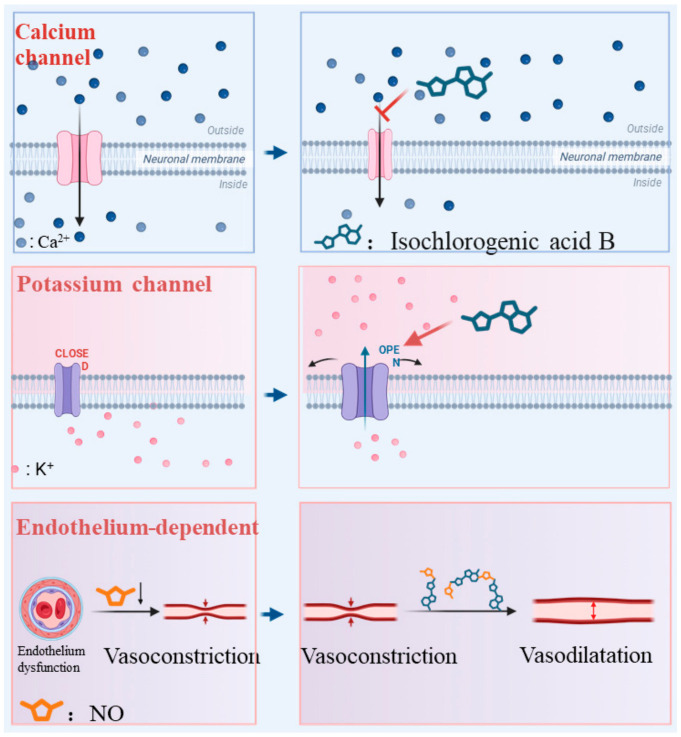
Potential mechanisms of isochlorogenic acid B in vasodilation via inhibiting VGCCs and ROCCs, activating K^+^ channels, and acting in an endothelial-dependent manner. The red T-bar denotes inhibition, whereas red arrows indicate activation.

L-NAME, a competitive NO synthase (NOS) inhibitor, reduces endothelial NO production and thereby suppresses NO-mediated vasorelaxation. Pretreatment with L-NAME (100 µM) attenuated the vasorelaxant effect of isochlorogenic acid B (Figure 6G), indicating its vasorelaxant action to be endothelium-dependent. Thus, the vasodilatory effects of isochlorogenic acid B are Ca^2+^ channel-dependent, activating K^+^ channels, and endothelium-dependent (Figure 7). Unlike classical vasodilators such as Ca^+^ channel blockers (e.g., nifedipine and nimodipine), which mainly inhibit Ca^2+^ influx, nitroglycerin induces vasodilation primarily through NO release. This multi-mechanistic regulatory profile may allow isochlorogenic acid B to exert effects under various pathological conditions [29].

## 3. Materials and Methods

### 3.1. Animals

Twenty male Sprague–Dawley (SD) rats, weighing 350–370 g, were purchased from Beijing Vital River Laboratory Animal Technology Co., Ltd. (License No. SCXK [Beijing, China] 2021-0006). The rats were housed in a standard animal facility under controlled conditions (temperature: 20–25 °C; relative humidity: 40–60%; 12 h light/dark cycle) and were provided access to food and water. All rats were allowed to acclimatize for 3 days prior to experiments. All animal experiments were conducted in compliance with the ethical standards approved by the Animal Research Committee of the China Academy of Chinese Medical Sciences (Approval No. 2022B175).

### 3.2. Instruments and Reagents

Instruments: ALC-M intestinal perfusion system (Shanghai Alcott Biotechnology Co., Ltd., Shanghai, China); DMT 620M microvascular tension measuring system (Aarhus, Denmark); data acquisition and analysis system (Power Lab, AD Instruments, Dunedin, New Zealand); dissection stereomicroscope (Leica S6E, Leica Microsy stems, Wetzlar, Germany); ultrasonic cleaner (Kunshan Ultrasonic Instruments Co., Ltd., Kunshan, China); circulating water vacuum pump (Zhengzhou Great Wall Scientific Industrial and Trade Co., Ltd., Zhengzhou, China); vacuum drying oven (Beijing Luxi Technology Co., Ltd., Beijing, China); thermostatic water bath (DK-S14, Senxin Laboratory Instrument Co., Ltd., Shanghai, China); UPLC I-Class ultra-performance liquid chromatography system (Waters Corporation, Milford, CT, USA); and Q-TOF high-resolution quadrupole time-of-flight mass spectrometer (Waters Corporation, Milford, CT, USA).

Reagents: LC-MS grade acetonitrile and formic acid (Thermo Fisher Scientific, Shanghai, China); isochlorogenic acid B (14534-61-3, Chengdu Manster Biotechnology Co., Ltd., Chengdu, China); KCl (7447-40-7, Alfa Aesar, Shanghai, China); U46619 (56985-40-1, MCE, Shanghai, China); L-NAME (56985-40-1, Sigma, St. Louis, MO, USA) and TEA (75-57-0, J&K Scientific Ltd., Beijing, China).

Krebs solution: NaCl (6.9544 g), KCl (0.3429 g), NaH_2_PO_4_ (0.1872 g), and NaHCO_3_ (1.2602 g) were dissolved in 500 mL of double-distilled water. MgCl_2_ (0.3736 g) and CaCl_2_ (0.1665 g) were dissolved in another 500 mL of double-distilled water. The two solutions were combined and glucose (1.0089 g) was added. The mixture was thoroughly shaken, and the PH was adjusted to 7.4.

Tyrode’s solution: KCl (0.28 g), NaH_2_PO_4_ (0.05 g), NaCl (8.0 g), MgCl_2_ (0.1 g), NaHCO_3_ (1.0 g), and glucose (1.0 g) were dissolved in 500 mL of double-distilled water. CaCl_2_ (0.2 g) was dissolved in another 500 mL of double-distilled water. The two solutions were mixed, shaken thoroughly, and freshly prepared before use.

Calcium-free Tyrode’s solution: Prepared in the same manner as Tyrode’s solution, except without CaCl_2_.

### 3.3. Preparation of XSB and Single-Herb IAS

According to the preparation process of XSB, 3.75 g of the total XSB extract (without excipients) and 3.75 g of each single-herb extract were each weighed, dissolved in 25 mL of Tyrode’s solution, ultrasonicated for 30 min, and filtered to obtain the corresponding extracts (150 mg/mL of the crude drug), which were used to prepare the IAS. The solutions were diluted to the required concentration prior to the experiment. The dosage of the IAS was expressed as the amount of crude drug equivalent used in its preparation.

Male SD rats were fasted overnight (12 h) and anesthetized with 4% isoflurane. A midline abdominal incision was made to expose the intestine, and four segments (14 cm each) were excised at 10 cm intervals distal to the pylorus. The segments were immediately placed on ice and carefully everted so that the serosal surface faced outward. One end of each intestinal segment was ligated, and 2 mL of Tyrode’s buffer was injected into the lumen. The other end of the segment was ligated, and the sacs were suspended in organ baths containing 25 mL of XSB or single-herb solutions. Control sacs were incubated in Tyrode’s buffer. The organ baths were continuously aerated with a gaseous mixture of 95% O_2_ and 5% CO_2_ and maintained at 37 °C. After 2 h of incubation, the sacs were removed for further analysis [10].

### 3.4. Preparation of Isolated Coronary Artery Rings

Rats were anesthetized with isoflurane, and their hearts were rapidly excised and placed in ice-cold Krebs solution. Septal branches of the coronary artery were dissected. The rat coronary artery can be approximately divided into four vascular segments, each about 2 mm in length. Two tungsten wires (diameter 40 um) were inserted through the lumen of each ring, which was then mounted in an organ bath and connected to an isometric force transducer for tension recording. The organ baths containing 5 mL of Krebs solution were maintained at 37 °C and continuously aerated with a mixture of 95% O_2_ and 5% CO_2_. The resting tension of each ring was gradually adjusted to the optimal level and allowed to equilibrate to establish a stable baseline.

Vascular reactivity was assessed by contracting the rings using KCl or U46619. After three washes to restore baseline tension, rings that produced contractions greater than 2 mN in response to KCl or U46619 were considered viable and used for further experiments [30]. Coronary arteries were randomly allocated to groups using a random number table to reduce selection bias.

### 3.5. Effects of XSB and Single-Herb IAS on Pre-Contracted Coronary Artery Rings

The vasorelaxant effects of XSB and single-herb IAS were evaluated using the coronary rings of rats. The precontraction induced by KCl or U46619 was defined as 100%. The vasorelaxation (%) was calculated as follows: (Precontraction-tension after drug)/Precontraction × 100%. Tissue contractility (%) = (control contraction-treatment contraction)/control contraction × 100%. Control contraction refers to the responses induced by cumulative KCl or U46619 without isochlorogenic acid B, serving as a reference to evaluate its inhibitory contractility effect. Drugs were added cumulatively into the organ baths containing the suspended coronary artery rings, with 3 min intervals between each addition.

### 3.6. Identification of the Components of Shan Zha IAS

#### 3.6.1. Sample Preparation

Acetonitrile (600 µL) was added to 300 µL of Shanzha IAS, vortexed for 5 min, and centrifuged at 12,000× *g* for 10 min. The supernatant was collected, vacuum dried, and redissolved in 100 µL of methanol. The solution was sonicated for 20 min, vortexed for 15 min, and recentrifuged. The supernatant was subjected to UPLC-Q-TOF-MS analysis.

#### 3.6.2. UPLC and MS Conditions

Chromatographic separation was achieved using a Waters UPLC HSS T3 column (2.1 mm × 100 mm, 1.8 µm). The mobile phases consisted of 0.1% formic acid in water (A) and 0.1% formic acid in acetonitrile (B), and the following gradient was used: 0–6 min, 2% B; 6–8 min, 2–16% B; 8–10 min, 16% B; 10–14 min, 16–47% B; 14–17 min, 47–48% B; 17–22 min, 48–54% B; 22–24 min, 54–56% B; 24–26 min, 56–78% B; 26–30 min, 78–95% B; 30–40 min, 95% B. The flow rate was 0.3 mL/min, the column temperature was 30 °C, and the injection volume was 3 µL. MS detection was conducted using an electrospray ionization (ESI) source in both positive and negative ion modes. The spray voltage was 3.5 kV (+) and 3.0 kV (−). Sheath gas flow was 35 arb, auxiliary gas flow was 10 arb, auxiliary gas temperature was 350 °C, and capillary temperature was 320 °C. Data were acquired in full MS/dd-MS^2^ mode over a *m*/*z* range of 50–1500, with resolution settings of 35,000 (MS^1^) and 17,500 (MS^2^). The normalized collision energy was set at 12–50 eV. A photodiode array detector was used to monitor the wavelength range of 150–500 nm.

#### 3.6.3. Compound Identification and Data Processing

A chemical database of Shanzha was established to facilitate the identification of the prototype constituents in the IAS. Compound names, exact molecular weights, and fragmentation patterns were retrieved from PubMed, Web of Science, and CNKI databases. UPLC-Q-TOF-MS was used to acquire accurate MS/MS datasets in the data-dependent acquisition mode. Compounds were identified by matching the experimental exact masses and fragmentation ions with those reported in the database, for chemical structure elucidation.

### 3.7. Molecular Docking

To systematically evaluate the binding potential of Shanzha IAS compounds to vasodilation targets, a high-throughput virtual screening workflow was established using the open-source molecular docking platform SailVina (https://github.com/beikwx/SailVina, accessed on 6 January 2026).

#### 3.7.1. Preparation of Structures of Small Molecules

2D structures (.sdf format) of the components were downloaded from PubChem (https://pubchem.ncbi.nlm.nih.gov/, accessed on 10 January 2026) and literature databases and batch converted into 3D docking format (.pdbqt) using Open Babel (V3.1.1). The pH was set at 7.0, and all stereoisomers were retained.

#### 3.7.2. Preparation of Target Proteins

Using “vasodilation’’ as the keyword, the core targets related to vasodilation were retrieved from the GeneCards database (https://www.genecards.org/, accessed on 15 January 2026). The top 50 targets ranked by relevance score were imported into the STRING database (https://string-db.org/cgi/input.pl, accessed on 15 January 2026) for protein–protein interaction (PPI) analysis. The species was set to “*Homo sapiens*”, the interaction confidence score threshold was set to medium confidence (>0.400), and the unconnected nodes in the network were hidden while other parameters were kept at default. The corresponding structural files were obtained from the RCSB Protein Data Bank (https://www.rcsb.org/, accessed on 20 January 2026) and uniformly converted into the “pdbqt” format. Protein preparation, including removal of water molecules, hydrogenation, and structural optimization, was performed using Auto Dock tools and the preprocessing utilities in SailVina.

#### 3.7.3. Identification of the Active Site and Preparation of the Docking Grid

Binding pockets were predicted using the P2Rank algorithm (Prank Web platform, https://prankweb.cz/, accessed on 20 January 2026), and the highest-scoring pocket was chosen as the docking region. In cases where no significant binding pocket was detected, a “whole-protein wrapping” mode was applied to cover all potential binding sites on the protein surface.

#### 3.7.4. High-Throughput Molecular Docking and Energy Scoring

Batch docking was performed using the following parameters: exhaustiveness = 8, num_modes = 10, and energy_range = 4 kcal/mol. Each compound was docked individually to each protein target, yielding a total of 300 docking results (50 compounds × 6 proteins). Binding affinity (kcal/mol) scores were extracted from the docking outputs and subsequently used for clustering analysis and heatmap visualization.

### 3.8. Mechanisms of Coronary Vasodilation Induced by Isochlorogenic Acid B

#### 3.8.1. Vasorelaxant Effects of Isochlorogenic Acid B

Isolated coronary artery rings of rats were pre-contracted using KCl (60 mmol/L) or U46619 (5 × 10^−7^ mol/L). Isochlorogenic acid B (5 × 10^−5^–2.5 × 10^−3^ mol/L) was added cumulatively at 3 min intervals, and relaxation responses were recorded to generate concentration–response curves. The control group was treated with equivalent volumes of dimethyl sulfoxide (DMSO). The vasorelaxation (%) was calculated as follows: (Precontraction-tension after drug)/Precontraction × 100%.

#### 3.8.2. Effects on Calcium Channels

After elution with calcium-free Tyrode’s solution, U46619 (5 × 10^−7^ mol/L) was used to induce contraction. Next, different concentrations of isochlorogenic acid B were added to observe its effects on vascular tension. The vasorelaxation (%) was calculated as follows: (Precontraction-tension after drug)/Precontraction × 100%.

Its effects on voltage-gated calcium channels (VGCCs) and receptor-operated calcium channels (ROCCs) were further investigated. VGCCs: After U46619-induced contraction and washout, the rings were incubated with isochlorogenic acid B (2.5 × 10^−3^ mol/L) for 10 min, followed by the cumulative addition of KCl. Tissue contractility (%) = (control contraction-treatment contraction)/control contraction × 100%.

ROCCs: After contraction induced by (60 mmol/L KCl) and washout, the rings were incubated with isochlorogenic acid B (2.5 × 10^−3^ mol/L) for 10 min, followed by the cumulative addition of U46619. Tissue contractility (%) = (control contraction-treatment contraction)/control contraction × 100%.

#### 3.8.3. Effects on K^+^ Channels and Endothelium-Dependent Relaxation

After vascular precontraction, TEA (1 × 10^−3^ mol/L) or L-NAME (1 × 10^−4^ mol/L) was added and incubated for 10 min. Contraction was induced with U46619 (5 × 10^−7^ mol/L). Subsequently, isochlorogenic acid B was added cumulatively, and the dose effect curves were drawn to evaluate its effects on K^+^ channels and endothelium-dependent relaxation. The vasorelaxation (%) was calculated as follows: (Precontraction-tension after drug)/Precontraction × 100%.

### 3.9. Date Processing and Statistical Analysis

All data were analyzed using GraphPad Prism 9.5.0. All data represent at least three independent biological replicates, expressed as mean ± standard deviation (SD). For data that conformed to normal distribution and homogeneity of variance, comparisons among multiple groups were performed using one-way analysis of variance (ANOVA), followed by Tukey’s multiple comparisons test for post hoc pairwise comparisons. If the data did not meet the assumptions of normality or homogeneity of variance, the Kruskal–Wallis H test was applied, followed by Dunn’s multiple comparisons test for post hoc analysis. *p* < 0.05 was considered statistically significant. Statistical significance was defined as * *p* < 0.05, ** *p* < 0.01, and *** *p* < 0.001 compared with the control group.

## 4. Conclusions

Traditional Chinese medicine (TCM) formulas have long been used in the treatment of cardiovascular diseases; however, the identification of their active constituents and underlying mechanisms remains a major challenge. Our research approach may provide insights into the identification of bioactive compounds within the herbal formula [30].

A combination of ex vivo pharmacological experiments, mass spectrometry component analysis, and molecular docking was used in this study to systematically elucidate the active material basis and underlying mechanisms of XSB. Shanzha was found to play the top contributing role in the vasorelaxation effect of XSB. This is the first study to report and validate the vasodilatory effect of isochlorogenic acid B from Shanzha. However, several limitations should be acknowledged. First, the cardiovascular pharmacological effects of isochlorogenic acid B need to be further verified in vivo. Second, investigations into the mechanism of action of isochlorogenic acid B relying solely on selective inhibitors are insufficient. Further experiments, such as patch-clamp, calcium imaging, NO measurement and NOS3 phosphorylation assay, are needed to definitively identify its vasodilatory mechanisms. In addition, although isochlorogenic acid B has been identified to exert vasodilatory activity, the combined effects of all the ingredients with vasodilatory activity in XSB still require further evaluation.

## Data Availability

The original contributions presented in this study are included in the article/Supplementary Materials. Further inquiries can be directed to the corresponding authors.

## Supplementary Materials

The following supporting information can be downloaded at: https://www.mdpi.com/article/10.3390/ph19050704/s1, Figure S1. Vasorelaxant activity evaluation of ACH (acetylcholine) and SNP (sodium nitroprusside) in 0.6, 2.5 and 10 uM. The results are presented as means ± SD (n = 6 coronary artery rings). Table S1. Prototype ingredients in Shanzha-IAS.

## Abbreviations

XSB	Xinshubao
IAS	Intestinal absorption liquid
VGCCs	Voltage-gated calcium channels
ROCCs	Receptor-operated calcium channel
NO	Nitric oxide
CO	Carbon monoxide
NPPC	Natriuretic peptide C
CRP	C-reactive protein
PTGIS	Prostaglandin I2 synthase
VEGFA	Vascular endothelial growth factor A
IL-6	Interleukin-6
