# Supplementary material for: Active Constituents and Mechanisms of Xinshubao Tablets in Coronary Vasorelaxation
Source: Pharmaceuticals (Basel). 2026 Apr 29;19(5):704. doi: 10.3390/ph19050704 (PMC13210279; doi:10.3390/ph19050704)
Supplement: Supplementary file 1 [file pharmaceuticals-19-00704-s001.zip › pharmaceuticals-4232773-supplementary/Figure S1.pdf]

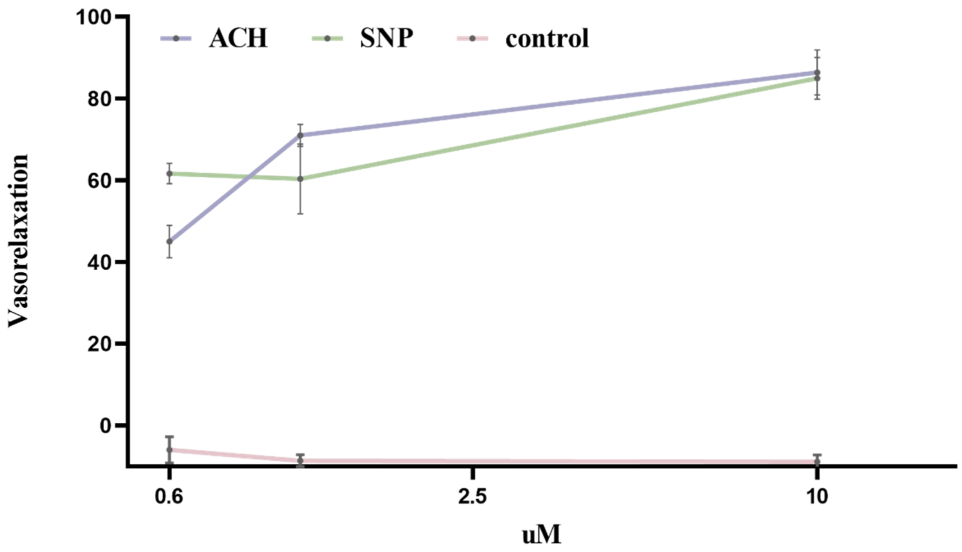

Figure S1. Vasorelaxant activity evaluation of ACH (acetylcholine) and SNP (sodium nitroprusside) in 0.6, 2.5 and 10uM. The results are presented as means ± SD (n = 6 coronary artery rings).
