# Supplementary material for: Active Constituents and Mechanisms of Xinshubao Tablets in Coronary Vasorelaxation
Source: Pharmaceuticals (Basel). 2026 Apr 29;19(5):704. doi: 10.3390/ph19050704 (PMC13210279; doi:10.3390/ph19050704)
Supplement: Supplementary file 1 [file pharmaceuticals-19-00704-s001.zip › pharmaceuticals-4232773-supplementary/Table S1.pdf]

Table S1. Prototype ingredients in Shan zha-IAS.

No.	Component name	RT (min)	Formula	Theoretical m/z	Observed m/z	Mass error (mDa)	Adducts	MS/MS
1	Quinic acid	0.51	$C_7H_{12}O_6$	191.0561	191.0568	0.7	[M-H]- /[M+CH3COO]-	101.0261, 111.0104, 131.0360, 149.0466
		0.52	$C_7H_{12}O_6$	193.0707	193.0706	-0.1	[M+H]+	175.0602, 157.0497, 129.0544, 111.0438, 99.0436
2	Citric Acid	0.55	$C_6H_8O_7$	193.0343	193.035	0.7	[M+H]+	145.0498, 139.0025, 129.0544, 127.0390
		0.56	$C_6H_8O_7$	191.0197	191.0204	0.7	[M-H]-	111.0104, 173.0102, 136.9892, 154.9994
3	Procyanidin C1	2.03	$C_{45}H_{38}O_{18}$	889.1951	889.1951	0	[M+Na]+	109.0281, 313.0891, 569.1679, 729.2053
		2.83	$C_{45}H_{38}O_{18}$	865.1985	865.195	-3.5	[M-H]-	381.1039, 497.1132, 647.1411, 263.0517

4	Procyanidin D1	2.67	$C_{60}H_{50}O_{24}$	1155.2764	1155.2765	0.1	[M+H]+	109.0281, 1075.2232, 633.1577, 313.0892
5	luteolin-3', 7-diglucoside	4.62	$C_{27}H_{30}O_{16}$	645.1227	645.1262	3.5	[M+Cl]-	179.0567, 183.0303, 381.1039, 209.0088, 283.0436
6	Procyanidin B2, (+)-	5.26	$C_{30}H_{26}O_{12}$	579.1497	579.1491	-0.6	[M+H]+	104.9920, 313.0887, 457.1308, 475.1414
7	Vanillic acid	7.69	$C_{8}H_{8}O_{4}$	169.0496	169.0499	0.3	[M+H]+	107.0491, 123.0441, 137.0593, 155.0317
8	Naringenin-5,7-O-diglucoside	7.7	$C_{27}H_{32}O_{15}$	595.1669	595.1671	0.2	[M-H]-	525.1468, 427.0886, 345.0825, 343.1245, 335.0641
9	Chlorogenic Acid	7.75	$C_{16}H_{18}O_{9}$	355.1023	355.1001	-2.2	[M+H]+	339.0997, 293.0662, 231.0832, 201.0454, 167.0311
		7.96	$C_{16}H_{18}O_{9}$	353.0878	353.0875	-0.3	[M-H]-	191.0565, 151.0024,

							/+CH3COO	225.0396, 231.0265
10	Caffeic Acid	8.15	$C_9H_8O_4$	239.0561	239.0567	0.6	+CH_3COO / [M-H]-	123.0466, 179.0374, 201.0181
		8.27	$C_9H_8O_4$	181.0496	181.0487	-0.9	[M+H]+	147.0433, 145.0255, 173.0593, 187.0582
11	Eucomic acid	8.15	$C_{11}H_{12}O_6$	239.0561	239.0567	0.6	[M-H]-	149.0616, 179.0374, 221.0457
		8.15	$C_{11}H_{12}O_6$	263.0526	263.0529	0.3	[M+Na]+	131.0493, 161.0597, 205.0488
12	3-O-Rhamnosyl quercetin	8.29	$C_{33}H_{40}O_{20}$	755.2040	755.2035	-0.5	[M-H]-	699.1706, 479.1762, 409.1353, 375.0920, 323.0982
13	Rutin	8.36	$C_{27}H_{30}O_{16}$	611.1606	611.1617	1.1	[M+H]+	595.1748, 387.0913, 175.0601, 129.0179
14	Isoschaftoside	8.47	$C_{26}H_{28}O_{14}$	565.1552	565.1542	-1	[M+H]+	339.1048, 295.0783, 313.0891, 405.1005

15	Neoisoschaftoside	8.47	$C_{26}H_{28}O_{14}$	565.1552	565.1542	-1	[M+H]+	339.1048, 295.0783, 313.0891, 405.1005
16	Sieboldin	8.61	$C_{21}H_{24}O_{11}$	453.1392	453.1402	1	[M+H]+	271.0790, 225.0739, 157.0497, 185.0433
17	Cinnamic acid	8.77	$C_9H_8O_2$	149.0597	149.0589	-0.8	[M+H]+	109.0283, 127.0390, 129.0544
18	Herbacetin	8.84	$C_{15}H_{10}O_7$	303.0499	303.0507	0.8	[M+H]+	133.0649, 243.0478, 271.0789
19	Cyanidin 3-glucoside	9	$C_{21}H_{21}ClO_{11}$	485.0845	485.0841	-0.4	[M+H]+	419.0959, 311.0739, 283.0797, 265.0291
20	Phlorizin	9.1	$C_{21}H_{24}O_{10}$	437.1442	437.1444	0.2	[M+H]+	370.1467, 163.0387
21	Epicatechin	9.12	$C_{15}H_{14}O_6$	335.0773	335.0775	0.2	[M+HCOO]-	125.0257, 161.0253, 133.0307
		9.63	$C_{15}H_{14}O_6$	291.0863	291.0841	-2.2	[M+H]+	147.0433, 177.0536, 249.1106, 303.0521

22	Quercetin 3-O-malonylglucoside	9.21	$C_{24}H_{22}O_{15}$	551.1032	551.1079	4.7	[M+H]+	475.0809, 253.069, 225.0732, 157.0496
23	Shikimic acid	9.22	$C_{7}H_{10}O_{5}$	173.0455	173.0465	1	[M-H]-	111.0104, 129.0570, 157.0516
		9.47	$C_{7}H_{10}O_{5}$	175.0601	175.0601	0	[M+H]+	157.0498, 111.0438, 161.0186
24	Procyanidin	9.34	$C_{30}H_{26}O_{13}$	593.1300	593.1287	-1.3	[M-H]-	287.0533, 439.1054, 375.0639
25	Kaempferol	9.47	$C_{15}H_{10}O_{6}$	287.0550	287.0527	-2.3	[M+H]+/[M+Na]+	161.0186, 111.0438, 157.0498, 233.0400
26	Isoquercitroside	9.58	$C_{21}H_{20}O_{12}$	463.0882	463.0885	0.3	[M-H]-/[M+HCOO]-	191.0565, 300.0281, 291.0083, 251.0154
27	Cyanidin 3-arabinoside	9.73	$C_{20}H_{19}ClO_{10}$	453.0594	453.0613	1.9	[M-H]-	255.0294, 246.0222
28	Ferulic Acid	9.91	$C_{10}H_{10}O_{4}$	217.0471	217.0487	1.6	[M+Na]+	121.0280, 163.0384, 235.0596, 205.0684

		10.3	$C_{10}H_{10}O_4$	193.0507	193.0511	0.4	[M-H]-	107.0515, 149.0616, 159.0486, 179.0358
29	3′-O-Arabinosyl-quercetin	10.3	$C_{20}H_{18}O_{11}$	433.0776	433.0796	2	[M-H]-	357.0574, 179.0358, 271.0241, 205.0138
30	Luteolin-7-O-glucoside	10.33	$C_{21}H_{20}O_{11}$	449.1078	449.1071	-0.7	[M+H]+	313.0802, 291.0837, 185.0429, 157.0494
31	Isochlorogenic Acid B	10.51	$C_{25}H_{24}O_{12}$	517.1341	517.1342	0.1	[M+H]+	391.0985, 225.0735, 163.0389, 211.0750
32	Vitexin -4''-O-glucoside	11.09	$C_{27}H_{30}O_{15}$	593.1512	593.149	-2.2	[M-H]-	247.0829, 289.0931, 431.1179, 525.1522
33	Dihydrocaffeic acid	11.16	$C_9H_{10}O_4$	181.0506	181.0513	0.7	[M-H]-	109.0315, 145.0309, 163.0400, 119.0516
34	bioquercetin	11.83	$C_{27}H_{30}O_{16}$	655.1516	655.1489	-2.7	[M+HCOO]-/[M-H]-	559.1507, 489.1314, 183.0305, 163.0619
35	Vitexin-4-rhamnosyl	11.95	$C_{26}H_{28}O_{14}$	565.1552	565.1529	-2.3	[M+H]+	279.0848, 271.0788, 189.0549, 207.0653

36	5-Hydroxyauranetin	12	$C_{20}H_{20}O_8$	389.1231	389.1193	-3.8	[M+H]+	283.0817, 237.0749, 207.0653, 161.0595, 149.0593
		12.76	$C_{20}H_{20}O_8$	387.1086	387.1097	1.1	[M-H]-	183.0303, 193.0520, 337.0786
37	Quercetin	12.38	$C_{15}H_{10}O_7$	301.0354	301.0352	-0.2	[M-H]-	151.0040, 145.0308, 193.0132, 287.0564
		12.38	$C_{15}H_{10}O_7$	303.0499	303.0507	0.8	[M+H]+	289.0706, 245.0440, 179.0315, 247.0602
38	Naringin	12.47	$C_{27}H_{32}O_{14}$	581.1865	581.187	0.5	[M+H]+	529.1318, 435.1239, 413.0861, 365.1054
39	Vitexin 2''-O-rhamnoside	12.54	$C_{27}H_{30}O_{14}$	579.1708	579.1723	1.5	[M+H]+	529.1318, 457.1281, 421.1076, 313.0881, 255.0869
40	Apigenin 7-β-D-glucopyranoside	13.51	$C_{21}H_{20}O_{10}$	491.1195	491.1196	0.1	[M+CH_3COO]-	161.0248, 313.0718, 401.0878, 353.0656

41	7-O-α-L-rhamnosyl-3-O-β-D-glucopyanosyl kaempferol	13.64	$C_{27}H_{30}O_{15}$	617.1477	617.1436	-4.1	[M+Na]+	525.1566, 457.1339, 403.0947, 337.0688, 109.0280
42	Maslinic acid	18.6	$C_{30}H_{48}O_4$	471.3480	471.3477	-0.3	[M-H]-/[M+HCOO]-	453.3372, 393.3160, 437.3066, 427.3577
43	Corosolic acid	18.88	$C_{30}H_{48}O_4$	495.3445	495.344	-0.5	[M+Na]+/[M+H]+	437.3406, 409.3458, 205.1584, 203.1790
44	p-Coumaric acid	21.83	$C_9H_8O_3$	165.0546	165.0545	-0.1	[M+H]+	77.0376, 104.1067
45	Oleic acid	25.61	$C_{18}H_{34}O_2$	281.2486	281.2486	0	[M-H]-	191.1451
46	Ursolic acid	26.35	$C_{30}H_{48}O_3$	455.3531	455.353	-0.1	[M-H]-/[M+HCOO]-	407.3314, 375.2693
		26.36	$C_{30}H_{48}O_3$	457.3676	457.3676	0	[M+H]+/[M+Na]+	439.3573, 411.3623, 393.3516, 205.1950, 203.1797, 191.1796
47	Betulin	27.11	$C_{30}H_{50}O_2$	443.3884	443.388	-0.4	[M+H]+	407.3669, 374.2674, 119.0853, 184.0734

48	Stearic acid	30.96	$C_{18}H_{36}O_2$	285.2788	285.2791	0.3	[M+H]+	257.2475, 167.1429
49	5,7,4′-Trihydroxyflavone 8-C-[β-D-glucopyranosyl (1-4)]-α-L-rhamnopyranoside	32	$C_{27}H_{30}O_{14}$	617.1268	617.1266	-0.2	[M+K]+	533.3602, 469.3287, 313.2732
50	Eugenol	34.16	$C_{10}H_{12}O_2$	165.0910	165.0914	0.4	[M+H]+	149.0961, 151.0751, 121.0647, 139.0751
